# Adaptation and psychometric evaluation of Hungarian version of the Fear of COVID-19 Scale

**DOI:** 10.1371/journal.pone.0261745

**Published:** 2021-12-29

**Authors:** Mona Stankovic, László Papp, Boglárka Nyúl, László Ivánkovits, Zoltán Pető, Annamária Töreki

**Affiliations:** 1 Department of Emergency Medicine, Albert Szent-Györgyi Health Centre, Faculty of Medicine, University of Szeged, Szeged, Hungary; 2 Department of Social Psychology, ELTE Eötvös Loránd University, Budapest, Hungary; National Cheng Kung University College of Medicine, TAIWAN

## Abstract

**Background:**

COVID-19 pandemic has had a global major healthcare, social and economic impact. In present study we aim to adapt the Fear of COVID-19 Scale to Hungarian.

**Materials and methods:**

Forward-backward translation method was used to translate the English version of the scale to Hungarian. Participants were a convenience sample of 2175 university students and employees. The study was conducted between January 18^th^ and February 12^th^ 2021. The test battery included Hungarian versions of Fear of COVID-19 scale, short Beck Depression Inventory (BDI-H) and State-Trait Anxiety Inventory (STAI).

**Results:**

The scale showed one-factor structure, the loadings on the factor were significant and strong (from .47 to .84). Internal consistency was very good (α = .84). Construct validity for the Fear of COVID-19 Scale was supported by significant and positive correlations with STAI (r = 0.402; p < 0.001) and BDI-H (r = 0.270; p < 0.001).

**Conclusion:**

The Hungarian version of Fear of COVID-19 Scale is a reliable and valid tool in assessing fear of coronavirus.

## Introduction

Over a year after the breakout of the COVID-19 epidemic–declared as global pandemic on March 11^th^, 2020 by the World Health Organization–coronavirus 2 (SARS-CoV-2) spread is still in uprise in several countries around the globe. At the time of writing of this paper globally 173.315.599 cases of coronavirus were reported and the infection caused in total 3.729.410 deaths. In Hungary alone there have been 806.008 reported cases and 29.854 deaths according to the Coronavirus Resource Center [1].

Vaccination against SARS-CoV-2 has started throughout 2020 and up until now in total 2.109.878.745 doses of vaccine were administered globally [1]. At the time of data collection in January of 2021, vaccination has just started in Hungary, and vaccines were accessible only to healthcare professionals. Since then, according to the official site of the Hungarian government 5.260.418 citizens received one (54% of the population) and 3.989.525 (41% of the population) received both doses of the vaccine [2]. However, even though Hungary has good vaccination rate for over a month, the number of daily new cases and death rates are decreasing slowly.

Globally 220 countries and territories have been affected by the pandemic of coronavirus and governments all around the world have been taking unprecedented measures in order to slow down the spread of the virus. Measures vary from border control, lockdown, contact tracing to public health measures such as physical distancing, self-isolation and handwashing [3]. The spread of coronavirus triggers feelings of depression, fear, stress and anxiety among general population as well as healthcare professionals [4–6]. The long-term effects of the COVID-19 related fear are in connection with decreased job satisfaction and performance as well as high levels of anxiety among healthcare personnel [6]. The burdens of the pandemic, such as social distancing, lockdown, quarantine or isolation [7], the long-term consequences, such as job loss, financial insecurities, disruption of daily activities [8, 9], together with the overestimation of death tolls [10] as well as sensationalistic news and broadcasting all amplify fears and often generate stigma [11–13]. To date there is no particular estimation as to the duration of the pandemic, which further deepens the feelings of uncertainty [14]. Negative psychosocial consequences of fear have been reported, during former epidemics establishing that people often oscillate between denial and phobia, while stigmatizing persons racially perceived as being the source of the infection [12, 15–18]. Fear is often accompanied with feelings of anxiety and depression, which additionally negatively impact one’s well-being and quality of life [13, 19–22].

According to Brooks [23], individuals kept in quarantine occasionally experience mental health issues, including anger, anxiety, confusion or PTSD [24–26]. Concurrently social isolation is strongly associated with anxiety and depression symptoms in both older and younger populations [13, 21, 27, 28]. Complementing medical treatments of coronavirus patients with psychological interventions would result in better-quality patientcare and overall better outcome for the entire population affected [13]. From the beginning of 2021 in some Hungarian COVID-19 inpatient facilities, clinical health psychologists have been a part of a medical team taking care of all COVID-19 infected patients. Colleagues working in these fields both help patients in coping with the effects of the infection and hospitalization, simultaneously relieving some burden of the medical staff.

Several measures have been created in order to assess the effect of coronavirus on mental health [29]. Ahorsu et al. [30] recently developed the Fear of COVID-19 Scale (FCV-19S), a measure adequate for assessment of the fear of coronavirus. This seven-item scale is a short and easy-to-use tool with very good internal consistency and concurrent validity positively correlating with measures of anxiety and depression [30, 31]. Since its development, the Fear of COVID-19 Scale has been translated and adapted to several languages [13, 14, 31–50]. The initial study examined the validity against the Hospital Anxiety and Depression Scale and Perceived Vulnerability to Disease Scale [30], similarly to the Portuguese [5], English [31], Italian [13], Arabic [46], Spanish [44] and Japanese [37] adaptations, while other studies confirmed the validity using various other measures of depression and anxiety [14, 32–35, 38, 39, 42, 43, 45, 47, 49, 50]. The Fear of COVID-19 Scale has high reliability across translations and cultural adaptations [4].

There is debate whether the Fear of COVID-19 Scale has a stable single-factor structure [4] as reported in the initial study [30] and several adaptations [5, 13, 14, 31, 32, 34, 36, 38–43, 46, 50–53] or if it is bi-factorial as reported in Paraguayan [44], Israeli [45], Chinese [47], Norwegian [49], Argentinian [33], Peruvian [35], Japanese [37] and Eastern European [54] populations. The two-factor models define a cognitive and somatic fear [49] or emotional and psychological [37] fear. Due to the high correlation between the factors Iversen [49] proposes a second-order hierarchical model with two latent factors (somatic and cognitive fear) serving as indicators of a second-order general fear of COVID-19 factor. According to the developers of the initial scale, items were designed to examine both types of fear responses (physical and psychological) [55] however they are confident in the scale’s single-factor structure [56, 57].

Lin et al. has investigated the invariance and psychometric properties of the Fear of COVID-19 Scale in the original and ten translated datasets and confirmed one-factor structure of the measure in different ethnic populations [56]. The study found the invariance to be supported across gender and age groups, but only partially across ethnic populations [56].

Adaptation of the Fear of COVID-19 Scale to Hungarian would be a useful tool for healthcare providers when in need to quickly assess an individual’s fear of coronavirus [30, 57, 58]. We aim to report psychometric properties, reliability qualities, concurrent validity and confirmatory validity of the Hungarian version of the Fear of COVID-19 Scale (FCV-19S) and examine factorial invariance across genders.

## Materials and methods

### Participants and procedure

Participants were a convenience sample of employees and students of the University of Szeged in Hungary. Participants were reached through the emailing system used at the university. Study announcements, containing brief information about the data collection and the questionnaire, the aim of the investigation as well as the webpage link to the study, were shared via email. All participants were at least 18 years of age. Answers to all questions were mandatory. Data was collected between January 18^th^ and February 16^th^, 2021 during which time potential participants got a weekly reminder. The online survey was prepared using the EvaSys Automation Software V7.1 (Electric Paper Evaluationssysteme GmbH, Germany) in compliance with all General Data Protection Regulations. A final sample comprising 2175 participants was used to validate the Hungarian version of the FCV-19S.

### Ethical considerations

The study was conducted with the permission of the Regional Medical and Research Ethics Committee of the University of Szeged (approval No.: 199/2020-SZTE). The study adhered to the guidelines of the Helsinki Declaration, 1975. Participation was voluntary and all participants were ensured in writing concerning the anonymity of the data as well as the nature and purpose of the data collection prior to consenting.

### Adaptation of FCV-19S into Hungarian

Adaptation was carried out in accordance with *Guidelines for the Process of Cross-Cultural Adaptation of Self-Report Measures* [59]. The forward-backward translation method was applied to adapt the FCV-19S into Hungarian. One psychologist, as a subject matter expert and two English lectors, experienced in English and Hungarian culture translated the original seven-item scale into Hungarian (forward-translation). The three Hungarian versions were then translated back to English by a second psychologist and two English translators none of whom have seen the original version of the scale (back translation). Finally, an expert panel of three members evaluated, scrutinized the translated versions, checked for cultural appropriateness and finalized the items. All seven questions were retained. The approved Hungarian translation was piloted among 20 people to examine the scale readability and potential ambiguity. No apparent problems were found during the pilot trial, no further changes were deemed necessary. The final Hungarian version of the Fear of COVID-19 Scale can be found in the S1 Appendix.

### Measures

#### Demographic information

Participants reported gender, marital status, number of children, highest level of education, if they have a chronic illness or mental illness diagnosis, if they regularly take any medication, whether they are a healthcare professional, if they underwent the COVID-19 infection and whether they plan to vaccinate themselves against COVID-19.

#### Hungarian Fear of COVID-19 Scale

The FCV-19S is a unidimensional, 7-item (e.g., “I am most afraid of coronavirus-19”) scale that measures one’s fear levels of COVID-19 [30]. In Hungarian adaptation of the scale, we have decided to use a four-point Likert scale for the ease of use with the rest of the measures. Compared to the original version of the measure, the four-point scale ranged from 1 (*strongly disagree*) to 4 (*strongly agree*) without the “neither agree nor disagree” option. The score ranges from 7 to 28 points and the higher score indicates greater fear of coronavirus-19. The original scale has shown robust psychometric properties including high internal consistency (α = .82) [30].

#### State-Trait Anxiety Inventory (STAI)

The Hungarian adaptation [60] of the State-Trait Anxiety Inventory (STAI) questionnaire was used to assess participant’s state anxiety [61]. The 20-item questionnaire was used on a 4-point Likert scale. Participants could reach a minimum of 20 and a maximum of 80 points. Higher score on scale represents higher state of anxiety. This screening tool is used widely in both non-psychiatric and clinical settings.

#### Beck Depression Scale (BDI-H)

Level of depression among participants was assessed using the shortened Hungarian version [62] of Beck Depression Scale [63], a nine-item questionnaire with a 4-point Likert scale. The shortened version is routinely used as equivalent to the original and the reliability of the Hungarian version is acceptable [64].

### Data analysis

Descriptive statistics were used to report the sample characteristics. Skewness, kurtosis and distributions of responses were analyzed with respect to each item. Internal consistency was assessed by Cronbach’s alpha coefficients (α), inter-item correlations and corrected item-total correlations. A Cronbach’s α of .70 or higher indicated acceptable reliability [65], minimum inter-item correlations between .15 and .50, and minimum corrected item-total correlations of .30 were used as indicators of internal consistency reliability. Concurrent validity was assessed by comparing the Spearman correlations between the FCV-19S and STAI and BDI-H results. These analyses were conducted using the IBM SPSS Statistics v 26 software.

A confirmatory factor analysis (CFA) was performed to investigate the proposed theoretical domain structure in the Hungarian sample using the mean- and variance-adjusted weighted least squares (WLSMV) estimator. Goodness of fit was assessed according to the following criteria: root mean square error of approximation (RMSEA ≤ .08); comparative fit index (CFI > .90 or more desirably ≥ .95); Tucker–Lewis index (TLI > .90) and chi-square.

To test for measurement invariance across gender, multiple group CFA analysis (MGCFA) was performed. Configural, metric and scalar invariance was examined, invariance was established if ΔCFI and ΔTLI ≤ −.01; ΔRMSEA < .015 as recommended by Chen [66]. Factor structure was conducted using the MPlus 8 Software [67, 68].

## Results

In total 2175 participants completed the survey, 1786 students and 390 employees. The participants were between 19 and 89 years of age (*M* = 28.59, *SD* = 10.75). Among the employees 255 (65.3%) still went to work, while 126 (32.3%) worked from home. In total 93 participants (23.8%) were healthcare workers, while 297 (76.2%) were not. Table 1. shows in depth further characteristics of the sample. All questions were mandatory, so there was no missing data.

**Table 1:** Sample characteristics (n = 2175).

Characteristics		Frequency (n)	%
Sex			
	Male	836	38.4
	Female	1339	61.6
Marital Status			
	Single	1448	66.6
	Married	319	14.7
	Living with a partner	350	16.1
	Divorced	50	2.3
	Widowed	8	0.4
Education			
	Vocational school or lower	165	7.6
	Highschool diploma	1086	49.9
	Higher educational qualification	57	2.6
	Bachelor	157	7.2
	Postgraduate (Master/PhD)	710	32.6
Living with chronic illness (yes)		339	15.6
Regularly taking medication (yes)		625	28.7
Previously diagnosed with mental illness (yes)		260	12.0
Previously diagnosed with COVID-19 (yes)		384	17.7
Already vaccinated (yes)		275	12.6

### Internal consistency and concurrent validity

The Cronbach’s α value for the Hungarian FCV-19S was .839, indicating a very good internal reliability. The inter-item correlations ranged between .41 and .60 and corrected item-total correlations varied between .59 and .68 indicating adequate internal consistency of the Hungarian FCV-19S. The skewness and kurtosis values presented in Table 2. suggest that at least items 3, 4, 6, and 7 are unlikely to be normally distributed, however all items were found to be reliable.

**Table 2:** Descriptive details for the FCV-19S.

Item	Factor loading	Mean (SD)	Skewness	Kurtosis	Cronbach’s α when deleted
1	.55	1.81 (0.78)	.744	.114	.810
2	.47	2.25 (1.01)	.164	-1.125	.832
3	.77	1.14 (0.46)	3.87	16.86	.823
4	.63	1.34 (0.66)	2.15	4.59	.813
5	.58	1.84 (0.88)	.785	-.215	.812
6	.82	1.11 (0.43)	4.46	21.92	.822
7	.84	1.21 (0.55)	3.06	9.94	.810

Overall Cronbach’s α = .84

Concurrent validity was supported by the significant correlations with the state anxiety and depression. Fear of COVID-19 significantly and positively correlated with STAI (r = 0.402; p < 0.001) and BDI-H (r = 0.270; p < 0.001).

Average variance extracted (AVE) and composite reliability (CR) were calculated. AVE was .56 while CR was .90 both of which are acceptable values.

### Confirmatory Factor Analysis (CFA)

Most items were distributed asymmetrically, with the highest frequencies in the lowest values. Analyzing the asymmetry and kurtosis of the seven items of the FCV-19S most of the items did not fall within the range of ± 1,5. Shapiro-Wilk normality test confirmed that all items were distributed in a non-normal way (p < .01). The Hungarian FCV-19S appeared to have a unidimensional structure: it had eigenvalue of 3.91 in a single factor model suggesting one factor as the optimal usable model.

Confirmatory factor analysis (CFA) was performed on the data based on the factor structure provided by Ahorsu et al. [30] in the original study. CFA is a multivariate statistical procedure, in which the number of factors and the relationship between measured and latent variables can be specified. Therefore, it is used to test the replicability of the original factor structure of a scale on a different sample or in a different cultural context. CFA also shows the goodness of fit of the examined model. Due to non-normality of the distribution of several ratings and the categorical nature of the data, we used the WLSMV estimator [67]. CFA was performed with MPlus 8 software [68]. Regarding the fit indexes, a satisfactory degree of fit of comparative fit index (CFI) and Tucker-Lewis Index (TLI) is close or higher than .95 [69]. The root mean square error of approximation (RMSEA) below .05 indicates excellent, around .08 adequate, and above .10 a poor fit. Standardized Root Mean Square Residual (SRMR) is an index of the average of standardized residuals between the observed and the hypothesized covariance matrices [66]. The value of SRMR indicates good fit under .05 and adequate around .08

A first order confirmatory factor analysis was ran to determine whether the factor structure of the original form of the scale would be confirmed in the Hungarian sample. The first model of the CFA revealed a poor fit for the seven-item single-factor construct (Table 3). CFI, TLI and RMSEA values were all above desirable thresholds.

**Table 3:** Results of the original and modified model fitting.

Model	χ2 (df)	CFI	TLI	RMSEA	90% CI
Original model	1139.64 (21)	.841	.789	.156	.149-.164
Modified model	325.56 (18)	.956	.932	.089	.080-.097

**Fig 1:**
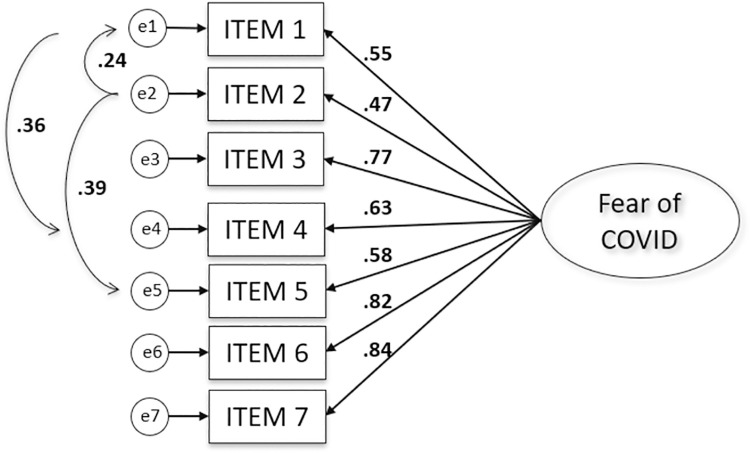
The results of the final CFA model of the Hungarian Fear of COVID-19 Scale.

The pattern of modification and inter-item correlations suggested the presence of notable error co-variance within the cluster of items #1 (I am most afraid of coronavirus-19) and #2 (It makes me uncomfortable to think about coronavirus-19), #1 and #4 (I am afraid of losing my life because of coronavirus-19) and #2 and #5 (When watching news and stories about coronavirus-19 on social media, I become nervous or anxious). When the error of variance of these items was correlated in the Model 2, the above-mentioned values decreased substantially and were below the required cut-off values. In the second model the goodness of fit values suggested a good fit and were the following: RMSEA = .089, CFI = .956, TLI = .932. Fig 1 presents an overview of the factor solution for the final model, including the factor loadings. All the paths shown in the model in Fig 1 were significant at the level of 0.01. Factor loadings of the items tested with CFA were found as I1 = .55, I2 = .47, I3 = .77, I4 = .63 I5 = .58, I6 = .82, I7 = .84. Since all factor loadings are greater than .30 these values can be considered adequate.

The factor structure was tested for measurement invariance (configural, metric and scalar) across gender (male and female) [69]. To test for measurement invariance across gender multiple group CFA analyses (MGCFA) was performed. The results showed a good fit for the model in both male (N = 836, χ^2^ = 65.972, df = 11, RMSEA = .077, CFI = .983, TLI = .967, SRMR = .029) and female (N = 1339, χ^2^ = 134.768, df = 11, RMSEA = .092, CFI = .967, TLI = .937, SRMR = .038) groups. The criteria for configural invariance (one-factor structure) were met. The comparison of the relative fit of the nested models showed that the criteria for metric invariance were also met, but not for the scalar invariance (Table 4), as indices diminished more than the recommended values (.01 for CFI and TLI; .015 for RMSEA) [66].

**Table 4:** Measurement invariance across gender.

Model	χ^2 ^(df)	CFI	TLI	RMSEA	90% CI	Δχ^2 ^(df)	ΔCFI	ΔTLI	ΔRMSEA
Configural	200.740 (22)	.974	.951	.086	.076-.098				
Metric	237.256 (28)	.970	.955	.083	.073-.093	36.516 (6)	-0.004	-0.004	-0.003
Scalar	381.416 (34)	.950	.938	.938	.088-.106	144.160 (6)	-0.020	-0.017	0.855

## Discussion

The primary aim of the present study was to examine psychometric properties of the Hungarian Fear of COVID-19 Scale (FCV-19S). Results suggest that the Hungarian version of the scale has a stable unidimensional structure, as reported in the original study [30] and several adaptations [13, 14, 31, 32, 34, 36, 38–43, 46, 50–53]. Across a large sample, the Hungarian Fear of COVID-19 Scale has good internal reliability and consistency.

We found the scale to be partially invariant with respect to gender. The measurement of the invariance across gender confirmed configural invariance, meaning that the basic factor structure is invariant across gender groups, men and women conceptualize the fear of coronavirus construct similarly. Metric invariance was also established, meaning that both genders respond to the items the identical way. The scalar invariance was not established. It refers to the condition that the level of the compared latent construct holds across groups [70]. When not supported it implies differential mean levels of the same latent construct between genders.

The significant correlation between the FCV-19S, STAI (level of state anxiety) and BDI-H (level of depression) confirms the concurrent validity of the scale. Similar correlations between FCV-19S and STAI have been formerly reported on the sample of Spanish university students [36]. Previous literature [4, 20, 22] supports the finding, that anxiety and depression are often comorbid with feelings of fear [19, 71], specifically fear during epidemics [15]. This relationship has been reported in several previous adaptations of the scale using various measures of anxiety and depression [13, 14, 31–39, 42–47, 49, 50] all reporting significant positive correlation between the scales.

The significant positive relationship measured between levels of fear and levels of state anxiety and depression, suggest that individuals who experience high levels of fear of coronavirus have also high probability to comorbidly be affected by these disorders [13]. This supports previous findings reporting that long periods of infectious epidemics are a breeding ground for development of psychophysical health issues and negative mental health conditions such as feelings of depression, anxiety, fear and phobias [12, 13, 15, 16, 18, 72–74]. The cross-sectional design prevents the causal interpretation of the data, so it is impossible to determine whether being depressed accelerates the fear of COVID-19 or whether the fear of coronavirus intensifies feelings of depression. Future longitudinal studies are needed to examine causes and consequences of the fear of coronavirus [13, 32].

Higher scores on FCV-19S predict higher scores on STAI and BDI-H, however, even though significant, the correlations between FCV-19S and STAI (r = .40) and between FCV-19S and BDI-H (r = .27) were moderate. This indicates a significant unshared variance between the FCV-19S, STAI and BDI-H suggesting that they represent more than one underlying construct, thus the Fear of COVID-19 Scale may provide some unique variance to the construct of overall anxiety and depression [50]. Additionally, while STAI focuses on individual’s state anxiety, their general experience of anxiety regardless of the source, FCV-19S is more specific, it focuses exclusively on their fear in regard to coronavirus.

The effect of the COVID-19 pandemic is not exclusively physical and psychological, but it raises severe systematic, social and economic issues. For instance, the spread of a viral infection is strongly in connection with the burden placed on the healthcare system and its possible overload [6] which negatively effects the healthcare professionals as well as the general population. Insecurity regarding the stability of the nation’s healthcare system, especially in poor public health contexts [13] increases stress levels among all citizens, which in turn can have a negative effect on both physical and mental health [8, 71]. Increased stress levels often result in higher prevalence of addictive behaviors (e.g., alcohol, tobacco or drug use) influencing person’s health and immune system, making them more susceptible to disease and infection [13]. Additionally, fear experienced during epidemics formerly often resulted in stigmatization of groups racially perceived as being the source of the infection causing public disturbances, scuffles, in extreme cases civil conflicts [13, 16, 17].

The Hungarian version of the Fear of COVID-19 Scale enables future research on causes and consequences of the fear of coronavirus and its effect on behaviors in connection to the pandemic. Secondly, it can be a direct tool used by the staff working in COVID-19 inpatient facilities for assessment of coronavirus-specific fear among patients [58].

These results come with limitations. Our sample is not representative of the Hungarian population. Even though the sample size is quite large, it is composed of university students and employees, thus it is not clear how this sample would generalize to Hungarian population. It would be useful to replicate the study on a representative sample.

Present study is cross-sectional, however the perception of COVID-19 pandemic is most likely fluctuating as we enter different stages of the epidemic globally and locally as well. For example, since data collection Hungary has had a stable vaccination rate, which could change the perception of the pandemic as well as the levels of fear associated with it. Likewise, present study design provides very little insight into causality. Future studies should include longitudional design to gain knowledge regarding causal relationship between fear of coronavirus, anxiety and depression.

In present study we investigated the psychometric properties of the Hungarian version of the FCV-19S with 2175 university students and employees. The psychometric testing of the Hungarian FCV-19S demonstrates that the measure is psychometrically robust and the final model shows a single-factor structure. It is a reliable and valid tool for assessing the severity of fear of COVID-19 among Hungarian adults.

## Supporting information

S1 Appendix (DOCX)
