# Supplementary material for: Adaptation and psychometric evaluation of Hungarian version of the Fear of COVID-19 Scale
Source: PLoS One. 2021 Dec 29;16(12):e0261745. doi: 10.1371/journal.pone.0261745 (PMC8716026; doi:10.1371/journal.pone.0261745)
Supplement: S1 Appendix — (DOCX) [file pone.0261745.s001.docx]

# S1 Appendix

Hungarian version of Fear of COVID-19 Scale

| 1 | Nagyon félek a koronavírustól. |
| --- | --- |
| 2 | Kellemetlen érzés a koronavírusra gondolnom. |
| 3 | Izzad a tenyerem, ha a koronavírusra gondolok. |
| 4 | Félek, hogy meghalok koronavírus fertőzésben. |
| 5 | Amikor híreket vagy történeteket látok a koronavírusról a közösségi médiában ideges leszek vagy szorongok. |
| 6 | Nem tudok aludni az aggodalomtól, hogy elkapom a koronavírust. |
| 7 | Gyorsabban dobog a szívem, ha a koronavírusra gondolok. |
